# Optimizing the wire pacing technique in transcatheter aortic valve replacement procedures: the experience of using J-wire pacing at a single center

**DOI:** 10.3389/fcvm.2025.1709297

**Published:** 2026-01-27

**Authors:** Ruisong Ma, Lili Zhang, Wang Liao, Sheng Wang

**Affiliations:** 1 Department of Cardiology, Hainan General Hospital, Haikou, China; 2 Department of Cardiology, Hainan Affiliated Hospital of Hainan Medical University, Haikou, China; 3 Department of Cardiology, Hainan Clinical Research Center for Cardiology, Haikou, China; 4 Department of Cardiology, Hainan Province Clinical Research and Cardiovascular Institute, Haikou, China; 5 Department of Cardiology, Hainan Province Clinical Medical Center, Haikou, China

**Keywords:** aortic stenosis, J-wire pacing, right ventricular pacing, traditional wire pacing, transcatheter aortic valve replacement

## Abstract

**Background:**

During transcatheter aortic valve replacement (TAVR) procedures, traditional wire pacing demonstrates safety and efficacy profiles similar to, or even superior to, right ventricular pacing. However, it still has disadvantages such as high thresholds and impedance and unstable pacing in some patients.

**Methods:**

Our center pioneered the following J-wire pacing technique: inserting a J-wire through the auxiliary access 8F sheath (for balloon-expandable valves) or the main access 20F sheath (for self-expanding valves) into the descending aorta and aligning it at the same height as the left ventricular wire creates a loop circuit between the J-wire and the left ventricular wire after the balloon or prosthesis has been inserted into the annulus. This study included a total of 26 patients. The impedance and threshold of traditional wire pacing and J-wire pacing were measured, and the pacing method with the lower threshold was selected as the intraoperative pacing method.

**Results:**

All 26 patients were assigned to the J-wire pacing group, achieving a 100% surgical success rate. The proportion of patients with a pacing threshold ≤5 V was significantly higher compared to traditional wire pacing (76.92% vs. 0%), while the proportion with a threshold ≥10 V was significantly lower (0% vs. 38.46%).

**Conclusions:**

J-wire pacing offers improved safety and effectiveness compared to traditional wire pacing in TAVR procedures. We herein share this single-center experience, hoping to provide novel insights for the refinement of TAVR procedures.

## Introduction

With the advancement of transcatheter aortic valve replacement (TAVR) techniques, the minimally invasive TAVR approach has been increasingly adopted by medical centers. Wire pacing is a crucial component of minimalist TAVR. It not only shortens procedure time, reduces fluoroscopy time, and lowers patient costs but also avoids the risk of pericardial tamponade associated with right ventricular (RV) pacing. Currently, there are three traditional wire pacing modalities. In all, the cathode is connected to the left ventricular (LV) wire via an alligator clip. The anode can be either (1) clipped to the skin incision of the femoral artery sheath, (2) clipped to a 5 mL syringe needle inserted through the skin at the groin, or (3) connected to a grounding pad adhered to the chest wall over the left ventricular apex. Extensive research has confirmed that traditional wire pacing techniques offer safety and efficacy comparable to RV pacing (1, 2). Recently, Yildirim et al. demonstrated that traditional wire pacing may be superior to RV pacing in terms of both safety and effectiveness (3). However, in clinical practice, we have observed that traditional wire pacing is associated with relatively high impedance and thresholds and, in some patients, it was unstable. Given that the maximum output voltage of temporary pacemakers is 20 V, some patients who undergo the aforementioned pacing methods cannot achieve pacing at voltages greater than twice their threshold, as required by clinical protocols (2), due to excessively high thresholds. This may lead to loss of capture during the procedure, potentially resulting in unacceptable complications such as displacement of the valve or balloon or damage to the valve leaflets.

## Methods

The inclusion criteria were based on the 2023 Chinese Clinical Practice Guideline for TAVR. The exclusion criteria included the following: (1) presence of pre-existing right bundle branch block, bifascicular block, or type II second-degree atrioventricular block; or (2) patients with pre-existing high-grade atrioventricular block or third-degree atrioventricular block who had not received a permanent pacemaker.

**Figure 1:**
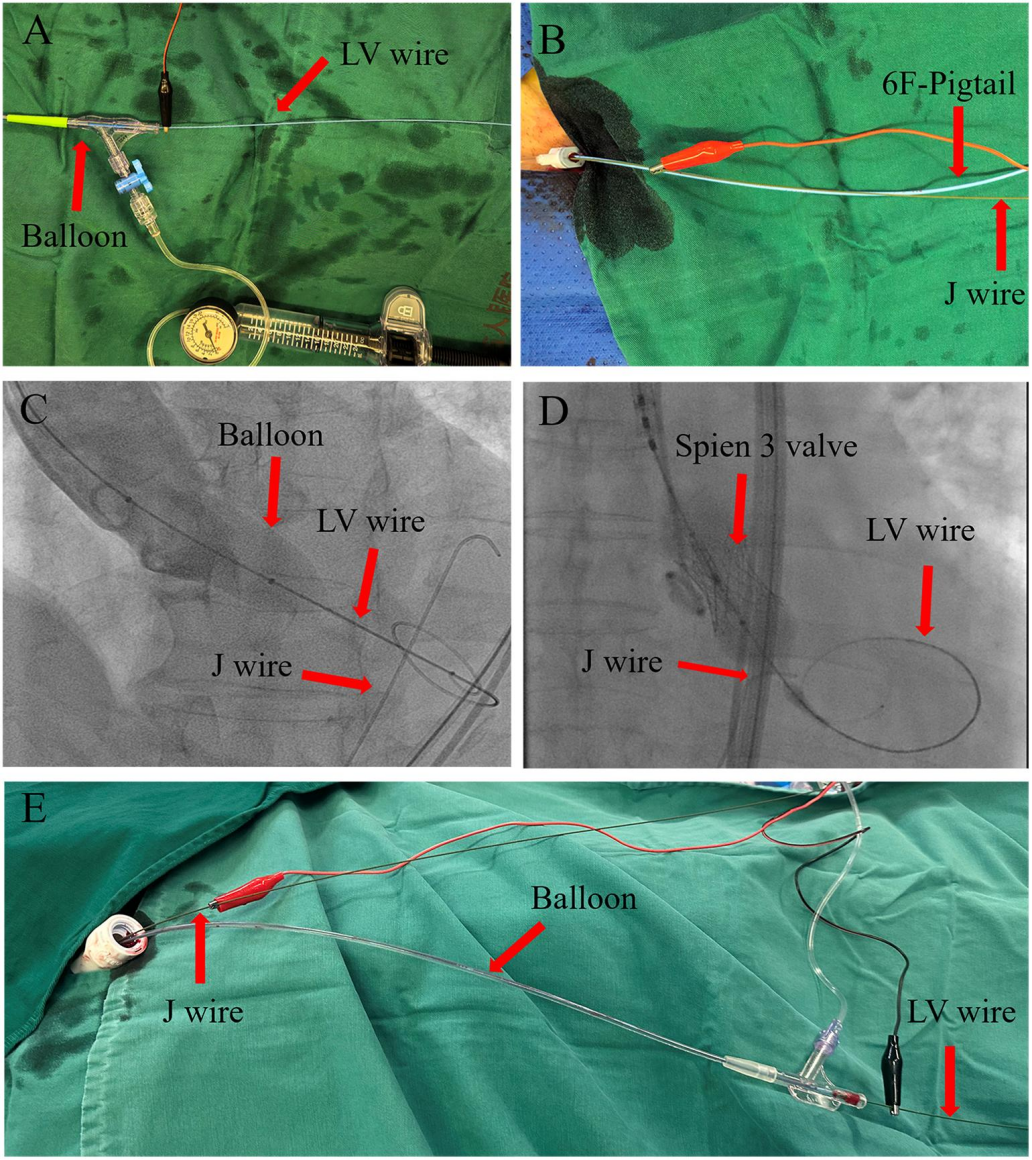
J-wire pacing procedure. **(A–D)** J-wire pacing in a balloon-expandable valve. After the balloon or prosthetic valve is advanced over the left ventricular (LV) wire to the aortic sinus, the J-wire is inserted through the auxiliary access 8F sheath and advanced to the descending aorta, with its tip aligned with the LV wire. The red alligator clip (anode) is connected to the J-wire and the black alligator clip (cathode) to the LV wire. **(E)** J-wire pacing in a self-expandable valve. After the balloon or prosthetic valve is advanced over the LV wire to the aortic sinus, the J-wire is inserted through the 20F sheath and advanced to the descending aorta, with its tip aligned with the LV wire. The red alligator clip (anode) is connected to the J-wire and the black alligator clip (cathode) to the LV wire.

Our center discovered that inserting a J-wire through the auxiliary access 8F sheath (for balloon-expandable valves; Figures 1A,B) or the main access 20F sheath (for self-expanding valves; Figure 1E) into the descending aorta and aligning it at the same height as the left ventricular wire (Figures 1C,D) creates a loop circuit between the J-wire and the LV wire (4) after the balloon or prosthesis has been inserted into the annulus. Pacing through this loop circuit significantly reduces the impedance and threshold of wire pacing, thereby expanding its applicability and enhancing its effectiveness and safety. We hereby share our experiences of this technique at this center. This study was approved by the Ethics Committee of Hainan Provincial People's Hospital (Approval No. EC-JS-2024-134-01) and written informed consent was obtained from all participants.

We pre-tested the threshold and impedance of the three traditional wire pacing methods and the J-wire pacing method. The pacing method with the lowest impedance and threshold was selected for intraoperative pacing; no randomization was performed, as the optimal method was chosen directly.

For the selected pacing method:

If the threshold was ≤5 V, a pacing voltage of 10 V was used during balloon dilation and valve deployment.
If the threshold was >5 V, a voltage twice the threshold value was used for pacing.
If the threshold was >10 V, right ventricular pacing was employed instead.

The primary endpoints were the safety and efficacy of the pacing. Efficacy was defined as stable and effective capture by the selected pacing method during balloon dilation and/or valve deployment, achieving a systolic blood pressure ≤60 mmHg with no loss of capture. Safety was determined by procedural safety and the occurrence of major adverse cardiovascular events (MACE) within 30 days.

Methods for measuring the threshold and impedance

Impedance measurement: Using the Medtronic 5,318 temporary pacemaker (range: 200 Ω–4,000 Ω), set the voltage to 5 V and the pacing rate to the patient's baseline heart rate plus 30 beats per minute (bpm). Measure the impedance. Values below 200 Ω are recorded as 200 Ω, and values above 4,000 Ω are recorded as 4,000 Ω.

Threshold measurement: Using the Medtronic 5,318 temporary pacemaker, start with a voltage of 10 V and decrease it in increments of 1 V. The pacing rate is set to the patient's baseline heart rate plus 30 bpm. The lowest voltage that achieves stable capture for at least 10 s is defined as the threshold.

Implementation of J-Wire pacing

After the balloon or prosthetic valve is advanced over the LV wire to the aortic sinus, position the J-wire in the descending aorta at the same height as the LV wire. Connect the anode to the proximal segment of the J-wire extracorporeal sheath and the cathode to the proximal segment of the LV wire extracorporeal sheath, thereby establishing the pacing loop circuit. Measure the threshold and impedance for this configuration. The J-wire is advanced after the balloon or prosthetic valve is positioned in the aortic sinus and is withdrawn before the balloon or prosthetic valve is removed (Figure 1).

### Statistical analysis

SPSS 20.0 was used for all the statistical analyses. Quantitative data are expressed as the mean ± SD or median ± quartiles. The Kolmogorov–Smirnov test was used to verify the normal distribution of the data. Student's paired *t*-test was used for between-group comparisons. One-way ANOVA was used to analyze comparisons among groups, and the least significant difference * post hoc* test or Tamhane's T2 test was used for multiple comparisons. The Kolmogorov–Smirnov test was used for comparisons between groups with non-normally distributed data. A *P*-value < 0.05 was considered statistically significant.

## Results

**Figure 2:**
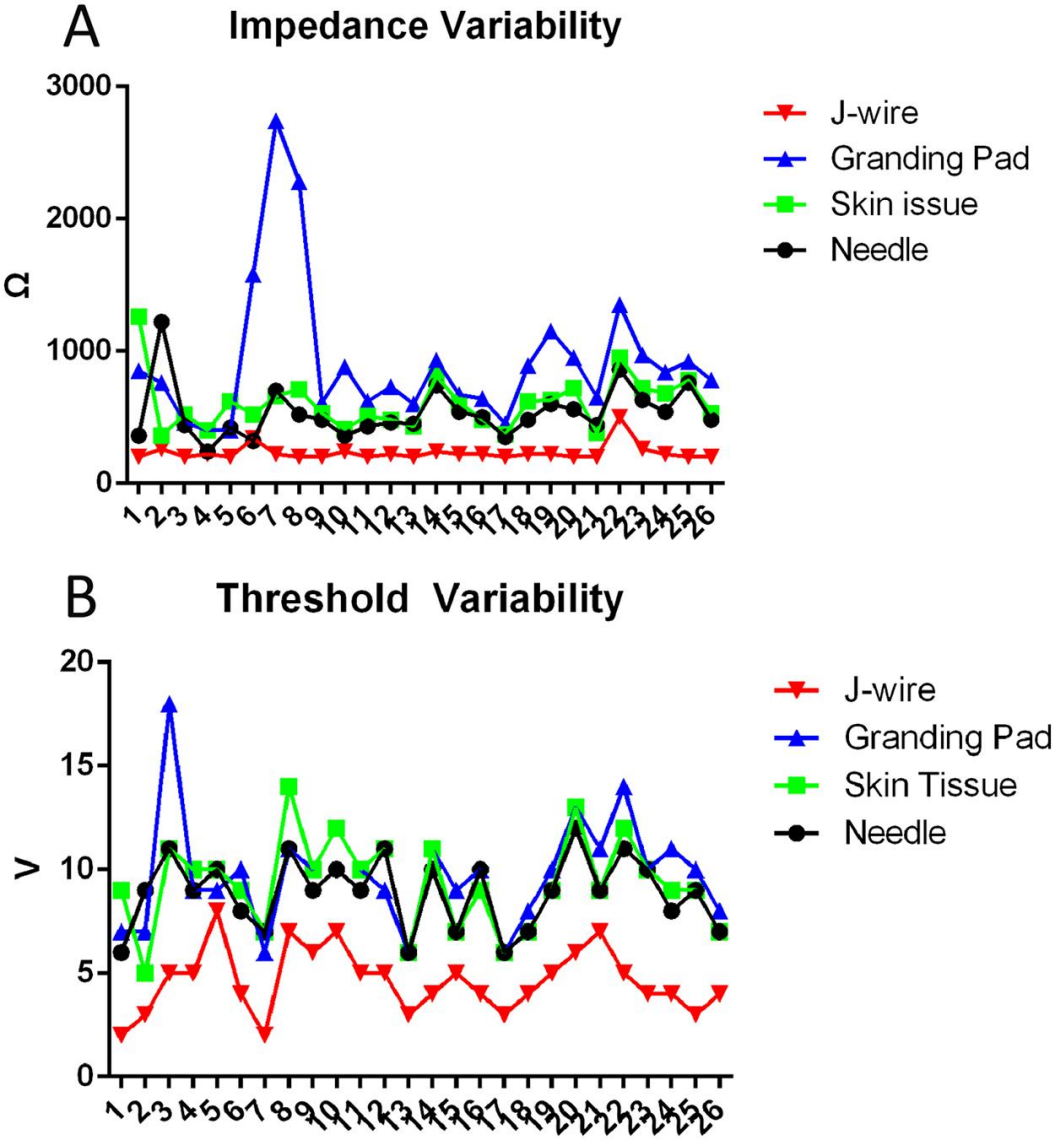
Impedance and threshold variability in different pacing methods. **(A)** Impedance variability in different pacing methods. J-wire pacing exhibits the lowest pacing impedance variability, while the ground pad shows the greatest impedance variability. **(B)** Threshold variability in different pacing methods. The threshold variability appears to be similar across all four methods.

Since June 2024, our center has performed a total of 26 TAVR procedures for aortic stenosis. J-wire pacing had lower impedance (200 ± 20 Ω, *P* < 0.05) and pacing thresholds (4.5 ± 1 V, *P* < 0.05) compared to the traditional wire pacing methods. The proportion of patients with a lower impedance (≤200 Ω, 46.15%, *P* < 0.05) and lower pacing threshold (≤5 V, 76.92%, *P* < 0.05) was significantly higher and the proportion of those with a higher threshold (≥10 V, 0%, *P* < 0.05) was significantly lower with J-wire pacing compared to traditional wire pacing. Moreover, the J-wire pacing demonstrated better pacing impedance homogeneity, with low impedance variability, but a similar threshold variability to those of the other three methods (Figure 2). All the patients (100%) ultimately underwent pacing using the J-wire pacing technique. The impedance and threshold values for the four wire pacing methods are presented in Table 1.

**Table 1:** The pacing threshold and impedance of J-wire pacing vs. traditional wire pacing, along with their distribution characteristics.

	Needle	Skin tissue	Grounding pad	J-wire
Impedance (Ω)	480 ± 110*	592.31 ± 194.68*	760 ± 360*	200 ± 20
Threshold (V)	9 ± 1.73*	9.35 ± 2.21*	10 ± 3*	4.5 ± 1
≤200 Ω ratio	0*	0*	0*	12 (46.15%)
>4,000 Ω ratio	0	0	0	0
≤5 V ratio	0*	0*	0*	20 (76.92%)
≥10 V ratio	10 (38.46%)*	12 (46.15%)*	15 (57.69%)*	0
Number and proportion of enrolled patients (Pcs and %)	0	0	0	26 (100%)

Needle: the anode was clipped to a 5 mL syringe needle that was inserted through the skin at the groin. Skin tissue: the anode was clipped to the skin incision in the femoral artery sheath. Grounding pad: the anode was connected to a grounding pad that was adhered to the chest wall over the left ventricular apex. J-wire: J-wire pacing.

* Compared with J-wire pacing, *P* < 0.05.

**Figure 3:**
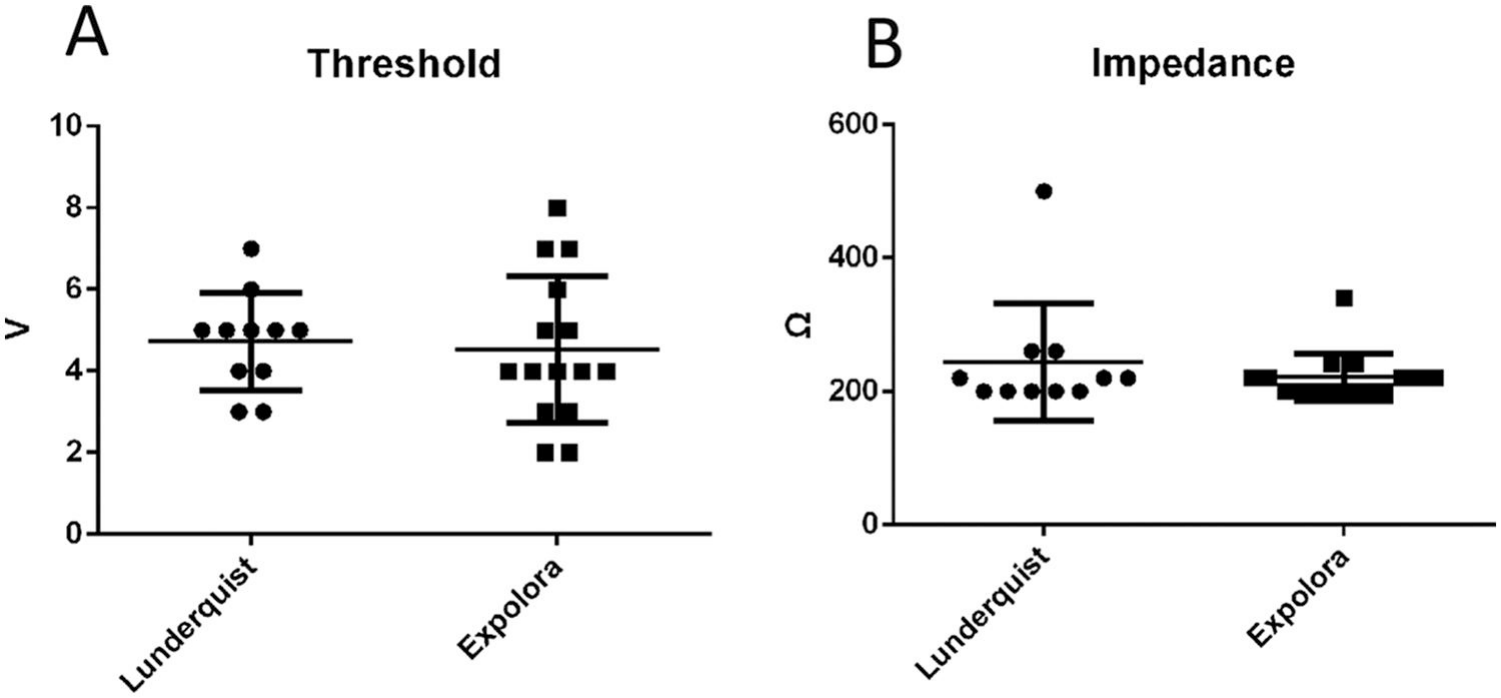
Threshold and impedance in the Lunderquist wire and Explora wire groups. **(A)** Thresholds in the Lunderquist wire and Explora wire groups. There was no significant difference between the groups. **(B)** Impedance in Lunderquist wire and Explora wire groups. There was no significant difference between the groups.

Patient baseline characteristics, procedural details, and 30-day MACE outcomes are listed in Table 2. Among these cases, self-expanding valves were used in 19 cases (73.08%) and balloon-expandable valves were used in seven cases (26.92%). Furthermore, the procedural success rate was 100% and one valve was implanted per patient. Moreover, the incidence of moderate-to-severe paravalvular leaks post-procedure, the incidence of pericardial tamponade, the rate of pacemaker implantation during hospitalization or within 30 days, and the 30-day heart failure readmission rate were all 0%. Finally, survival at 30 days was 100%. A subgroup analysis revealed no significant differences in impedance and threshold between the Lunderquist wire (William Cook European Aps, Denmark) and the Explora wire (Peijia Medial (Suzhou) Co., Ltd, China) groups (Figure 3). One patient developed a new-onset small-area intracerebral hemorrhage and was discharged on postoperative day 22 following TAVR after treatment. After TAVR, the transaortic max gradient significantly decreased (21.88 ± 8.52 mmHg vs. 106.35 ± 77.25 mmHg, *P* < 0.05), hemoglobin showed a slight decrease (107.42 ± 19.35 g/L vs. 119.69 ± 21.00 g/L, *P* < 0.05), and NT-proBNP exhibited a downward trend, though it did not reach statistical significance (1,213 ± 3,226.5 vs. 3,327.5 ± 7,298, *P* = 0.061). There were no significant differences in creatinine levels or left ventricular ejection fraction (LVEF).

**Table 2:** Patient baseline characteristics, procedural details, and 30-day MACE outcomes.

Characteristic	J-wire pacing
Number/percentage	26 (100%)
Age (years)	72.81 ± 8.16
Sex	
Male	18 (69.23%)
Female	8 (30.77)
Body mass index	22.59 ± 5.11
Hypertension	16 (61.54%)
Diabetes	8 (30.77%)
Atrial fibrillation	1 (3.85%)
Chronic kidney disease	5 (19.23%)
Coronary artery disease	6 (23.08%)
Stroke	5 (23.08%)
Previous aortic valve replacement	0
Previous pacemaker	0
Creatinine (pre-op vs. post-op), μmol/L	103.08 ± 78.70 vs. 104.31 ± 107.88
Hemoglobin (pre-op vs. post-op), g/L	119.69 ± 21.00 vs. 107.42 ± 19.35*
LVEF (pre-op vs. post-op)	58.92 ± 9.00 vs. 60.46 ± 7.76
Transaortic max gradient, mmHg (pre-op vs. post-op)	106.35 ± 77.25 vs. 21.88 ± 8.52*
NT-proBNP (pre-op vs. post-op), ng/L	3,327.5 ± 7,298 vs. 1,213 ± 3,226.5
SAPIEN 3 valve	7 (26.92%)
Taurus Elite valve	15 (57.69%)
Venus A valve	4 (15.38%)
LV wire: Lunderquist	11 (42.31%)
LV wire: Explora	15 (57.69%)
Efficacy of pacing stimulation	26 (100%)
Pre-dilation	22 (88%)
Post-dilation	8 (30.77%)
One prosthetic heart valve device implanted	26 (100%)
Procedural success	26 (100%)
Postprocedural paravalvular leak	
None	8 (30.77%)
Trivial	8 (30.77%)
Mild	10 (38.46%)
Incidence of pericardial tamponade	0
J-wire access site vascular complications	0
30-day heart failure readmission rate	0
Rate of temporary or permanent pacemaker implantation within 30 days	0
Incidence of 30-day stroke	1
Incidence of 30-day myocardial infarction	0

LVEF, left ventricular ejection fraction; LV, left ventricular.

* *P* < 0.05.

## Discussion

This study demonstrates that the J-wire pacing technique is straightforward and technically simple. In our preliminary studies, we found that positioning the J-wire in the aortic arch or aortic sinus did not reduce its pacing impedance or threshold (4). Instead, it carried risks of aortic injury and interference with valve deployment. Therefore, we opted to place the J-wire in the descending aorta at the same height as the left ventricular guidewire. Compared to traditional wire pacing methods, it retains their established advantages while effectively overcoming key limitations. First, it significantly reduces the pacing threshold and impedance. The intraoperative pacing voltage was maintained at least twice the pacing threshold, a requirement that could not be consistently met with traditional guidewire pacing due to its inherently high threshold. This protocol thus fully guaranteed pacing efficacy, thereby mitigating the risks of leaflet laceration, valve displacement, or procedural failure resulting from loss of capture, expanding the applicability of wire pacing, and enhancing its safety and efficacy. The lower impedance and threshold observed with J-wire pacing compared to traditional guidewire pacing may be attributed to the closer proximity of the J-wire to the left ventricular guidewire, along with the effective electrical isolation provided by the balloon or valve delivery system and the arterial sheath between the two wires. A subgroup analysis indicated no significant difference in impedance or threshold between the Lunderquist wire and the Explora wire groups, suggesting that the type of guidewire had no notable impact on these parameters. Additionally, the surgical success rate was 100% in both groups. Second, J-wire pacing avoids the risk of tissue injury associated with subcutaneous needle puncture or clamping of subcutaneous tissue with alligator clips. Third, it eliminates involuntary muscle contractions around the puncture needle or alligator clips during pacing. Traditional wire pacing demonstrates similar or even superior safety and efficacy profiles compared to right ventricular pacing. Right ventricular pacing not only leads to a higher risk of groin hematoma and cardiac tamponade but also prolongs both procedural and fluoroscopy times, increasing the financial burden on patients. Tjong et al. (5) reported a vascular complication incidence of 2.0% and cardiac perforation incidence of 1.6% in patients who underwent right ventricular pacing. Multiple studies have confirmed a higher vascular and cardiac perforation complication rate in right ventricular pacing than in LV pacing (3). Due to ethical considerations, this study did not directly compare J-wire pacing with RV pacing nor did it establish a randomized control group. Instead, it directly compared J-wire pacing with conventional guidewire pacing and selected the method with the lowest threshold and impedance as the preferred pacing approach. All 26 patients (100%) were assigned to the J-wire pacing group and the procedural success rate was 100%. J-wire pacing may demonstrate superior safety and efficacy compared to traditional wire pacing methods. Consequently, when compared to RV pacing, J-wire pacing is likely non-inferior, or potentially even superior, in terms of both safety and efficacy.

## Limitations

This study confirms the safety and efficacy of J-wire pacing. However, the following limitations should be acknowledged: (1) Single-center experience with limited sample size: This report represents a single-center experience with a small number of cases and larger, multi-center studies are needed to validate these findings; (2) Lack of randomized comparison to RV pacing: This study did not employ a randomized controlled design comparing J-wire pacing directly to RV pacing and randomized controlled trials are required to definitively establish the comparative safety and efficacy of J-wire pacing vs. RV pacing; (3) Sheath size limitation for balloon-expandable valves: In cases using balloon-expandable valves, the 14F femoral sheath could not simultaneously accommodate both the valve delivery system and the J-wire. Therefore, the auxiliary access sheath had to be upgraded to an 8F sheath to facilitate J-wire insertion; and (4) Potential for sheath oozing: In some patients, predominantly those receiving balloon-expandable valves, the slow oozing of blood from the hub of the arterial sheath was observed after J-wire insertion. To mitigate this, we minimized the time the J-wire remained in the descending aorta by withdrawing it after balloon or valve positioning and immediately prior to balloon or delivery system removal.

## Data Availability

The raw data supporting the conclusions of this article will be made available by the authors without undue reservation.
